# Association of Tau Pathology With Clinical Symptoms in the Subfields of Hippocampal Formation

**DOI:** 10.3389/fnagi.2021.672077

**Published:** 2021-07-14

**Authors:** Xinting Ge, Dan Zhang, Yuchuan Qiao, Jiong Zhang, Junhai Xu, Yuanjie Zheng

**Affiliations:** ^1^School of Information Science and Engineering, Shandong Normal University, Jinan, China; ^2^Laboratory of Neuro Imaging (LONI), USC Stevens Neuroimaging and Informatics Institute, Keck School of Medicine, University of Southern California, Los Angeles, CA, United States; ^3^School of Medical Imaging, Xuzhou Medical University, Xuzhou, China; ^4^College of Intelligence and Computing, Tianjin Key Lab of Cognitive Computing and Application, Tianjin University, Tianjin, China

**Keywords:** Alzheimer’s disease, hippocampal subfield, tau pathology, PET imaging, behavior symptoms

## Abstract

**Objective**: To delineate the relationship between clinical symptoms and tauopathy of the hippocampal subfields under different amyloid statuses.

**Methods**: One hundred and forty-three subjects were obtained from the ADNI project, including 87 individuals with normal cognition, 46 with mild cognitive impairment, and 10 with Alzheimer’s disease (AD). All subjects underwent the tau PET, amyloid PET, T1W, and high-resolution T2W scans. Clinical symptoms were assessed by the Neuropsychiatric Inventory (NPI) total score and Alzheimer’s Disease Assessment Scale cognition 13 (ADAS-cog-13) total score, comprising memory and executive function scores. The hippocampal subfields including Cornu Ammonis (CA1–3), subiculum (Sub), and dentate gyrus (DG), as well as the adjacent para-hippocampus (PHC) and entorhinal cortex (ERC), were segmented automatically using the Automatic Segmentation of Hippocampal Subfields (ASHS) software. The relationship between tauopathy/volume of the hippocampal subfields and assessment scores was calculated using partial correlation analysis under different amyloid status, by controlling age, gender, education, apolipoprotein E (*APOE*) allele ɛ4 carrier status, and, time interval between the acquisition time of tau PET and amyloid PET scans.

**Results**: Compared with amyloid negative (A−) group, individuals from amyloid positive (A+) group are more impaired based on the Mini-mental State Examination (MMSE; *p* = 3.82e-05), memory (*p* = 6.30e-04), executive function (*p* = 0.0016), and ADAS-cog-13 scores (*p* = 5.11e-04). Significant decrease of volume (CA1, DG, and Sub) and increase of tau deposition (CA1, Sub, ERC, and PHC) of the hippocampal subfields of both hemispheres were observed for the A+ group compared to the A- group. Tauopathy of ERC is significantly associated with memory score for the A- group, and the associated regions spread into Sub and PHC for the A+ group. The relationship between the impairment of behavior or executive function and tauopathy of the hippocampal subfield was discovered within the A+ group. Leftward asymmetry was observed with the association between assessment scores and tauopathy of the hippocampal subfield, which is more prominent for the NPI score for the A+ group.

**Conclusion**: The associations of tauopathy/volume of the hippocampal subfields with clinical symptoms provide additional insight into the understanding of local changes of the human HF during the AD continuum and can be used as a reference for future studies.

## Introduction

Alzheimer’s disease (AD) is characterized by the deposition of pathologic amyloid and tau proteins (Marks et al., 2017; Gordon et al., 2019; Scharre, 2019). Compared with β-amyloid (Aβ) plaques, tau deposition has been found to have a stronger association with cognitive decline during the AD continuum (Brier et al., 2016). Specimen research has revealed that the arise of tau deposition is firstly found in the trans-entorhinal cortex and then spreads into the temporal lobe regions such as the hippocampal formation (HF), and finally reaches the neocortex (Braak and Del Tredici, 2011; Braak et al., 2011). As the central node of the mnemonic circuitry, impairment of the HF has received much attention from other researchers (Yushkevich et al., 2015; Adler et al., 2018; Evans et al., 2018) to study the occurrence and progression of AD.

The HF has been associated with memory and cognitive functions and is conventionally used as one of the early biomarkers of several neuropsychiatric disorders including AD and Parkinson’s disease (PD; Ikram et al., 2010; Adler et al., 2018; Das et al., 2019). AD subjects present a significant decrease in hippocampal volume and multifaceted impairment of adult hippocampal neurogenesis (AHN) compared to cognitively normal (CN) individuals (Adler et al., 2018; Moreno-Jiménez et al., 2019). However, morphometry changes of the whole HF may be insufficient to delineate the detailed neurodegenerative features during the progression of AD as the human HF is comprised of several substructures including the dentate gyrus (DG), the Cornu Ammonis (CA), the subiculum (Sub), and the associated white matter tracts (Naidich et al., 1987). Subfields of the HF may exhibit differential patterns in their association with cognitive performance in specific domains, as well as the subsequent risk of dementia. The atrophy of subiculum and pre-subiculum regions were found strongly associated with executive dysfunction (Evans et al., 2018). CA1 and fimbria also showed the trend toward significant volume reduction with the progression of AD (Parker et al., 2019; Zhao et al., 2019). In addition, the impairment of the HF can be regarded as the neurodegeneration assessment following the amyloid/tau/neurodegeneration (AT[N]) framework (Jack et al., 2016, 2019; Jack Jr et al., 2018). The spreading patterns of biomarker findings such as tau deposition of the hippocampal subfields, as well as its association with the clinical symptoms during the AD continuum, however, remain relatively underexplored.

In addition, behavioral changes are the accompanying characteristics with AD progression and severely impact the patients’ quality of life and caregivers’ burdens (Zhao et al., 2016; Deb et al., 2018). CN individuals with abnormal behavior symptoms were found to be at higher risk of developing mild cognitive impairment (MCI; Mok et al., 2004; Masters et al., 2015). Much research has demonstrated that the presence of mild behavioral impairment can be used as an “at-risk” state for cognitive decline and dementia (Ismail et al., 2016; Yoon et al., 2019). As one of the early markers of AD, impairment of the hippocampal subfield may also be associated with the rise of behavior symptoms. The characterization of these relationships will help us to determine if the presence of behavior symptoms provides additional information for the understanding of the AD continuum.

The specific aim of the current study was to observe the relationship between tau deposition of each hippocampal subfield and clinical symptoms (assessed by the memory, cognition, executive function, and behavior scores) under different amyloid status (amyloid negative and positive). The Alzheimer’s Disease Assessment Scale-Cognitive (ADAS-cog) was used to measure the cognitive performance (Petersen et al., 2010; Kueper et al., 2018) and the Neuropsychiatric Inventory (NPI) was used to quantify the severity and frequency of behavior symptoms in the ADNI project (Cummings, 1997; Nunes et al., 2019). The volume of each hippocampal subfield was also assessed in the current research to calculate its association with the clinical scores. AD-related factors including age, gender, education, and apolipoprotein E (*APOE*) allele ε4 carrier status were considered in the statistical analysis based on previous studies (Tosun et al., 2017; McCartney et al., 2018). We hypothesize that the tauopathy/volume of different hippocampal subfields may have diverse association patterns with the clinical symptoms, even under the same amyloid status. They may exhibit an enhanced association between the tauopathy/volume of hippocampal subfield and the clinical symptoms with the presence of amyloid pathology. Our results may provide additional insight into the detailed analysis of the local changes of the human HF during the AD continuum and can be used as the reference for future AD studies.

## Materials and Methods

### Participant

ADNI project was conducted to measure the progression of MCI and early AD based on serial MRI, PET, other biomarkers, and clinical and neuropsychological assessments (Weiner et al., 2015). The diagnostic criteria in ADNI was described beforehand and informed written consent was obtained from all participants at each site (Petersen et al., 2010). In our study, we firstly screened subjects who underwent both ^18^F-AV-1451 PET and structural T1 scans in the latest visit. Subjects with amyloid florbetapir (AV-45) PET scans and T2 scans (High-resolution hippocampus sequence) within the time interval of 1 year before/after the acquisition time of tau PET scans were then selected. The Aβ status was determined by previous studies with a cutoff of 1.11 for AV-45 tracer (Landau et al., 2014). Participants with age >65 years and complete cognitive and behavioral assessments were included in our study as we focused on late-onset AD. By June 11th of 2019, 143 participants meeting the above requirements were selected from ADNI-2 and ADNI-3 (Table 1).

**Table 1:** Data characteristics (A−: amyloid negative, A+ : amyloid positive, n.s.: no significant, *p* < 0.05).

Amyloid status	A−	A+	*P*-value
Number of subjects (*N*)	88	55
CN/MCI/AD	59/28/1	28/18/9
Age (years)	75.05 ± 6.77	77.99 ± 7.90	0.0192
Education (years)	16.40 ± 2.68	16.47 ± 2.73	n.s.
Gender (M/F)	40/48	24/31	n.s.
*APOE* ɛ4 (0/1/2)	71/15/1	25/22/7	1.96e-06
MMSE	28.75 ± 1.74	26.55 ± 4.35	3.82e-05
Memory	0.82 ± 0.69	0.31 ± 0.98	6.30e-04
Executive function	0.94 ± 0.95	0.33 ± 1.24	0.0016
ADAS-cog-13	14.23 ± 6.02	19.58 ± 10.10	1.33e-04
NPI	2.86 ± 6.40	5.07 ± 8.94	n.s.

### T1-Weighted and T2-Weighted MRI Acquisition and Processing

All subjects were scanned by 3.0 T MRI scanners using a 3D MP-RAGE or IR-SPGR T1-weighted sequences and high-resolution hippocampus T2-weighted sequence. The detailed protocol can be found online^1^.

### Tau PET Image Acquisition and Processing

Tau PET images were preprocessed according to the standardized protocols at each ADNI site. All images were verified with quality control and processed with realignment, averaging, and resampled to an isotropic voxel size of 8 mm. We obtained the brain ROIs based on the Desikan-Killiany Atlas (Desikan et al., 2006) and mapped the PET image to the structural T1-weighted MRI image. Standardized uptake value ratio (SUVR) images were calculated for each subject using the whole cerebellum gray matter as the reference region.

### Segmentation of the Hippocampal Subfields

**Figure 1:**
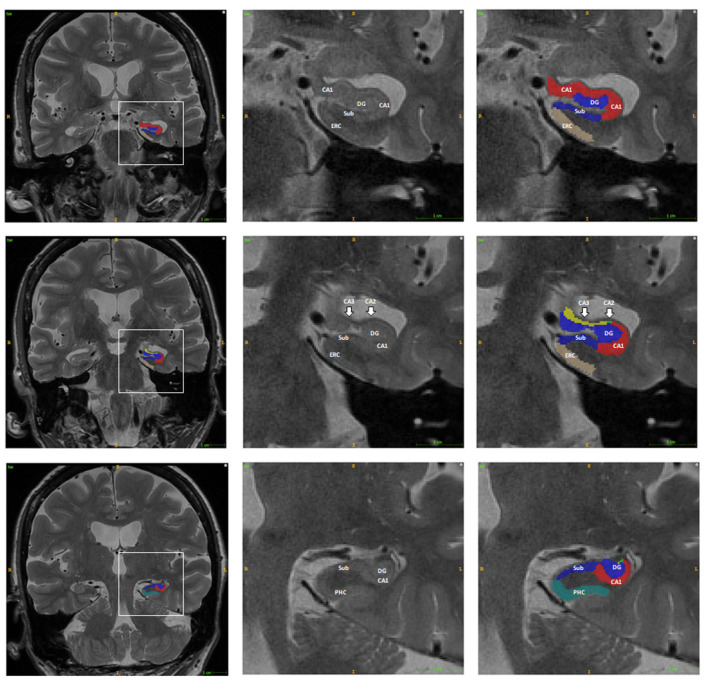
Example of the automatic segmentation of the hippocampal subfield using ASHS software. The boundary of each hippocampal subfield of the hippocampal head (first row), body (second row), and tail (third row) are shown using ITK-SNAP software. The right two columns are the enlargement of the box in the left column. Abbreviations: DG, dentate gyrus; CA, Cornu Ammonis; Sub, subiculum; PHC, para-hippocampus; ERC, entorhinal cortex.

ASHS (Automatic Segmentation of Hippocampal Subfields) software was used for the automatic segmentation of the hippocampal subfields for each subject^2^. T1 weighted and high-resolution T2 weighted MRI data were imported into the ASHS to automatically parcellate the HF and adjacent brain regions into CA1, CA2, CA3, Sub, para-hippocampus (PHC), entorhinal cortex (ERC), and DG (Figure 1). The ERC and PHC were also considered in the current study as these two regions were adjacent to the HF and were closely related to the progression of AD. The CA2 and CA3 regions were considered together in the following analysis as the size of these regions were relatively small. Mean SUVRs of the six regions on each hemisphere were finally calculated from the SUVR images.

### Clinical Symptoms

In the current research, we focus on the behavior score and the cognition score, as well as the comprised memory and executive function scores to delineate their relationship with tau pathology. The total score of NPI based on 12 domains was used to assess the behavioral symptoms. The total score of ADAS-cog based on 13 domains was used to measure cognitive symptoms. The composite score for memory was composed of scores of the Rey’s Auditory Vocabulary List Test (RAVLT), Alzheimer’s Disease Assessment Scale—cognitive subscale-11 (ADAS-Cog), Logical Memory (LM), and MMSE recall scores. The composite score for executive function included Category Fluency (animals and vegetables scores), Trail Making Test (TMT) A and B, Digit span backward, Wechsler Adult Intelligence Scale-Revised (WAIS-R) Digit Symbol Substitution, and five Clock drawing items (circle, symbol, numbers, hands, time). Clinical scores were acquired within the time interval of 3 months before/after the acquisition time of Tau PET scans.

### Statistical Analysis

For paired group comparison based on amyloid status (A+ and A−), a two-tailed Student *t*-test was applied to the mean SUVR or volume of each hippocampal subfield between A− and A+ groups. For hemispheric comparison, a two-tailed Student *t*-test was applied to the mean SUVR or volume of each hippocampal subfield between the left and right hemisphere under different amyloid status.

To assess the association of clinical symptoms and mean SUVR (or volume) of each hippocampal subfield, partial correlation analysis was conducted on two groups (A+ and A−) for each hippocampal subfield. The NPI total score, ADAS-cog-13 total score, comprised memory score, and comprised executive function score were treated as the response variable and the mean SUVR or volume of each hippocampal subfield as the predictor. Age, gender, education, *APOE* allele ε4 carrier status, and the time interval between the acquisition time of amyloid PET and tau PET scans were used as the covariates of the partial correlation analysis. For all statistical tests, the false discovery rate (FDR) correction was applied for the correction of multiple comparisons. An adjusted *p*-value less than 0.05 was considered as statistically significant in all analyses.

### Data Availability

All ADNI data used in our experiments are publicly available through LONI IDA^3^.

## Results

### Data Characteristics

As we can see from Table 1, no significant difference in education level and gender distribution are found between the A− and A+ groups. Compared to the A− group, the A+ group has older subjects (*p* = 0.0192), more *APOE* allele ε4 carriers (*p* = 1.96e-06), and is more impaired based on the MMSE (*p* = 3.82e-05), memory (*p* = 6.30e-04), executive function (*p* = 0.0016), and ADAS-cog-13 scores (*p* = 5.11e-04).

**Figure 2:**
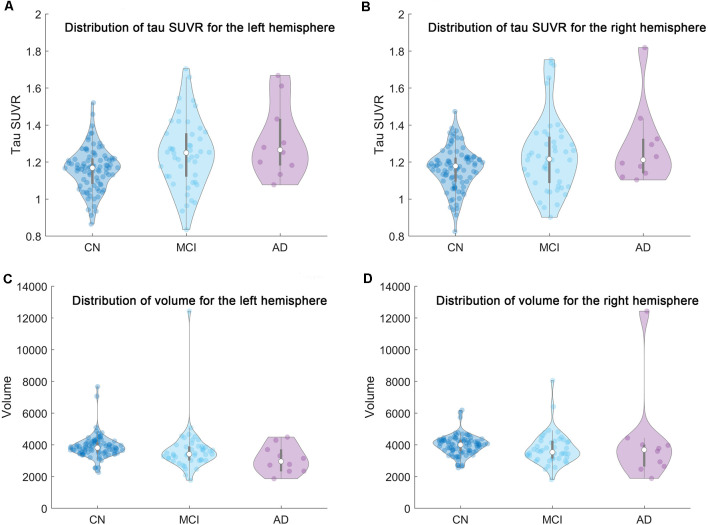
The distribution of the mean tau SUVR and total volume of the whole region comprised of the six subfields in the current research for each group (CN/MCI/AD) were shown based on violin plot. From left to right and top to bottom are the distribution of **(A)** the mean tau SUVR for the left hemisphere, **(B)** the mean tau SUVR for the right hemisphere, **(C)** the total volume for the left hemisphere, and **(D)** the total volume for the right hemisphere, respectively.

The distribution of the mean tau SUVR and total volume of the whole region comprised of the six subfields in the current study were plotted in Figure 2. We should note that there may be several outliers in the current cohort. Outliers were then removed from each group based on the mean tau SUVR and total volume before the statistical analysis was conducted. If the outlier criterion was satisfied for any condition (mean SUVR or total volume for left or right hippocampi), this subject would be excluded as an outlier. These calculations were performed using the “isoutlier” function of MATLAB with the “mean” method. After outlier removal, three subjects in the amyloid negative group and one subject in the amyloid positive group were removed from the statistical analysis steps.

### Comparison of Volume and Tau Deposition of the Hippocampal Subfields Between Amyloid Negative and Positive Groups

Significant volume decreases of the CA1, Sub, and DG for both hemispheres are observed from the A− group to the A+ group. Tau deposition of the CA1, Sub, ERC, and PHC for the A+ group increase significantly in both hemispheres compared to the A− group (Table 2).

**Table 2:** Volume and tau deposition of the hippocampal subfields between different amyloid status (*p* < 0.05, FDR correction. n.s.: not significant).

	Hemisphere	CA1	DG	Sub	ERC	PHC	CA2/CA3
Volume	Left	0.0036	0.0414	0.0414	n.s.	n.s.	n.s.
	Right	0.0037	0.0037	0.017	n.s.	n.s.	n.s.
SUVR	Left	0.0110	n.s.	0.0027	0.0001	0.0014	n.s.
	Right	0.0417	n.s.	0.0006	0.0001	0.0006	n.s.

### Hemispheric Differences of the Hippocampal Subfields Within Specific Amyloid Status

For A− group, no significant difference is found between the left and right hippocampal subfield for tau SUVR. Hemispheric differences were prominent for hippocampal volume (leftward lower) mainly in the CA1 (*p* = 0.0069), DG (*p* = 0.0026), PHC (*p* = 0.0026), and CA2/CA3 (*p* = 1.92e-08) regions.

Similarly, no significant difference is found between the left and right hippocampal subfield for tau SUVR within the A+ group. The hemispheric difference is also prominent for hippocampal volume (leftward lower) mainly in the PHC (*p* = 0.0055) and CA2/CA3 (*p* = 2.57e-08) regions.

### Partial Correlations Between Tau Deposition or Volume of the Hippocampal Subfields and Clinical Assessment Scores for A− Subjects

As can be seen from Table 3, the tau SUVR is found significantly correlated with memory score in the ERC for the left hemisphere, while no significant correlation was observed between the other clinical scores and tau SUVR of the hippocampal subfields for either hemisphere.

**Table 3:** Associations between assessment scores and tau SUVR of the hippocampal subfields for A- group (*p* < 0.05, FDR correction).

Hemisphere	Assessment score	CA1	DG	Sub	ERC	PHC	CA2/CA3
Left	Memory	0.54	0.62	0.32	**0.04**	0.54	0.62
	ADAS-cog−13	0.49	0.49	0.18	0.18	0.35	0.49
	Executive function	0.47	0.47	0.47	0.47	0.47	0.47
	NPI	0.53	0.53	0.53	0.53	0.53	0.53
Right	Memory	0.67	0.81	0.52	0.051	0.67	0.81
	ADAS-cog-13	0.78	0.78	0.34	0.30	0.34	0.72
	Executive function	0.39	0.39	0.31	0.31	0.35	0.39
	NPI	0.30	0.30	0.30	0.30	0.30	0.30

*Bold values mean the *p*’s < 0.05 and statistically significant*.

Differently, the volume of the left Sub and CA1 are significantly correlated with memory score, while no significant correlation is observed between the other assessment scores and hippocampal volumes (Table 4).

**Table 4:** Associations between assessment scores and volume of the hippocampal subfields for A− group (*p* < 0.05, FDR correction).

Hemisphere	Assessment score	CA1	DG	Sub	ERC	PHC	CA2/CA3
Left	Memory	**0.01**	0.053	**0.004**	0.16	0.96	0.96
	ADAS-cog-13	0.07	0.43	0.07	0.61	0.96	0.48
	Executive function	0.50	0.86	0.09	0.86	0.86	0.48
	NPI	0.92	0.92	0.88	0.92	0.56	0.92
Right	Memory	0.27	0.21	0.21	0.21	0.31	0.78
	ADAS-cog-13	0.55	0.55	0.55	0.55	0.89	0.55
	Executive function	0.99	0.96	0.39	0.39	0.39	0.99
	NPI	0.51	0.51	0.51	0.67	0.51	0.67

*Bold values mean the *p*’s < 0.05 and statistically significant*.

### Partial Correlations Between Tau Deposition or Volume of the Hippocampal Subfields and Clinical Assessment Scores for A+ Subjects

As we can see from Table 5, significant correlations between the assessment scores and tau SUVR are found in the Sub, ERC, and PHC for the left hemisphere. Tau SUVR of CA1 region is also significantly associated with ADAS-cog-13 and NPI scores for the left hippocampi.

**Table 5:** Associations between assessment scores and tau SUVR of the hippocampal subfields for A+ group (*p* < 0.05, FDR correction).

Hemisphere	Assessment score	CA1	DG	Sub			CA2/CA3
Left	Memory	0.08	0.64	**0.01**	**0.003**	**0.003**	0.20
	ADAS-cog-13	**0.02**	0.31	**0.001**	**0.0005**	**0.0005**	0.72
	Executive function	0.37	0.90	**0.048**	**0.008**	**0.008**	0.51
	NPI	**0.04**	0.25	**0.03**	**0.03**	**0.03**	0.83
Right	Memory	0.26	0.74	0.059	**0.03**	**0.02**	0.45
	ADAS-cog-13	0.06	0.51	**0.004**	**0.002**	**0.009 **	0.84
	Executive function	0.43	0.74	0.11	0.054	**0.03**	0.74
	NPI	0.20	0.27	0.20	0.27	0.20	0.82

*Bold values mean the *p*’s < 0.05 and statistically significant*.

As to the right hemisphere, the correlations with tau SUVR are found in the ERC and the PHC with memory score, in the Sub, ERC, and PHC with ADAS-cog-13 score, and in the PHC with executive function score. No significant correlation is found between the NPI score and tau SUVR of the right hippocampal subfields.

Significant correlations between the volumes and memory score are found in all the subfields for the left hemisphere, while the association is only found with the CA1, ERC, and CA2/CA3 regions for the right hippocampi. The volume of the CA1, DG, ERC, and CA2/CA3 are found significantly correlated with the NPI score for the left hemisphere. However, there is no significant correlation between the volume of the left subfields with either ADAS_cog_13 or executive function score. Differently, the ADAS_cog_13 score is found to be significantly correlated with the volume of the ERC and the executive function score is significantly correlated with the volume of the DG and ERC for the right hemisphere. The NPI total score is found associated with the volume of the CA1, Sub, and ERC for the right hippocampi (Table 6).

**Table 6:** Associations between assessment scores and volume of the hippocampal subfields for A+ group (*p* < 0.05, FDR correction).

Hemisphere	Assessment score		DG	Sub		PHC	CA2/CA3
Left	Memory	**0.008**	**0.008**	**0.03**	**0.008**	**0.02**	**0.04**
	ADAS_cog_13	0.14	0.23	0.11	0.11	0.74	0.68
	Executive function	0.16	0.12	0.19	0.35	0.12	0.83
	NPI	**0.008**	**0.008**	0.06	**0.005**	0.25	**0.008**
Right	Memory	**0.04**	0.053	0.053	**0.0004**	0.39	**0.04**
	ADAS_cog_13	0.09	0.17	0.09	**0.02**	0.91	0.15
	Executive function	0.10	**0.03**	0.12	**0.03**	0.12	0.12
	NPI	**0.07**	0.12	**0.01**	**0.003**	0.39	0.06

*Bold values mean the *p*’s < 0.05 and statistically significant*.

## Discussion

Atrophy of the HF is one of the early biomarkers for the diagnosis of neurodegenerative disorders including AD (Pizzi et al., 2016). However, neurodegeneration such as changes of the HF is not specific to AD and several neuropsychiatric disorders may have the same outcomes (Das et al., 2019). With the proposed AT[N] framework, the combination of the neurodegeneration assessed by the decrease of cortical thickness or the atrophy of HF and AD-related biomarkers such as pathology of amyloid and tau proteins were used to distinguish the stages of the AD continuum (Jack et al., 2016). In the current study, the memory score was found significantly associated with tau pathology in the ERC of the left hemisphere compared to the other hippocampal subfields, even for the amyloid negative individuals. On the contrary, impairment of cognition, behavior, and executive function were related to the changes of tauopathy/volume of hippocampal subfields when the amyloid status became positive. The impairment patterns of the hippocampal subfields observed in the current research are essential for the understanding of the AD spectrum and can be used as a reference for future AD studies.

Pathology of amyloid and tau proteins are the two defining hallmarks that can characterize the progression of AD. The presence of abnormal amyloid status was regarded as the ‘disease state’ to determine if the subject is during the Alzheimer’s pathologic process (Brier et al., 2016). Individuals with positive amyloid biomarkers demonstrated a higher risk of the conversion from cognitive normal to mild cognitive impairment (Donohue et al., 2017). There is a strong association of elevated tau deposition in both medial temporal lobe structures and the neocortex with positive amyloid status across the normal aging to clinical dementia (Marks et al., 2017). In the current study, higher tau deposition is discovered in the CA1, Sub, ERC, and PHC of both hemispheres for the A+ group as compared to the A− group (Table 2). Significant volume decreases of the CA1, DG, and Sub of both hemispheres are also observed from the A- group to the A+ group. All confirm the increased disease severity with the presence of elevated amyloid pathology.

Previous studies showed that tau pathology in the medial temporal lobe (MTL) is a key driver of memory impairment in AD and is an important biomarker for neurodegeneration (Marks et al., 2017; Scott et al., 2020). Repeated tau PET scans have been an effective measurement to track the disease progression, while amyloid PET is more responsible for the detection of the early Alzheimer pathology (Hanseeuw et al., 2019). Tau deposition in the temporal lobe has been a better predictor of cognitive decline than the emergence of amyloid plaques in any region of the brain (Brier et al., 2016). The crucial role of tau deposition is recommended as a candidate target for AD therapy to deal with the limitations of the amyloid cascade hypothesis (Giacobini and Gold, 2013). We thus focused on the association of tau pathology with clinical symptoms in the hippocampal subfields under different amyloid statuses to delineate the detailed progression of AD. Tau SUVR of the ERC showed a significant correlation with the comprised memory score for the A− subjects in the current research (Table 3). ERC is one of the most vulnerable regions for the deposition of tau tangles and is closely associated with memory function (Braak et al., 2011). Impairment of the ERC has been regarded as an essential marker for the classical analysis of AD. Our results confirmed that the ERC should be paid more attention for the subsequent studies.

With the presence of amyloid pathology, the correlation between the tau SUVR with assessment scores spread into several subfields (Table 5). The tau SUVR of the Sub, ERC, and PHC are correlated with the comprised memory score, ADAS-cog-13 score, executive function score, and NPI total score for the left hippocampi. The tau SUVR of the CA1 region is also found significantly associated with the ADAS-cog-13 score and NPI total score for the left hemisphere. For the right hippocampi, the comprised memory score and ADAS-cog-13 score are found significantly correlated with the deposition of tau tangles in the ERC and PHC. It is known that the spatiotemporal spread of tau tangles follows a stereotypical trajectory starting from the locus coeruleus and the trans-entorhinal cortex, and then extending to the ERC, the HF, and finally the neocortex (Fuster-Matanzo et al., 2018). The associations between the tau deposition of the hippocampal subfields and assessment scores in the current research demonstrate that the development of AD may be affected in a progressive manner from the ERC and the PHC to the Sub, and eventually to the CA1 region on a smaller scale. On the other hand, the DG and the CA2/CA3 regions are less affected by the tau pathology. The DG is one of the few areas in the mammaliasn brain in which new excitatory neurons are continuously generated throughout life. The neural plasticity that results from the continuous integration of newly born excitatory granule cells may contribute to the DG network activity and withstand tau deposition. This could finally slow the disease progression (Tuncdemir et al., 2019; Christian et al., 2020). In addition, the NPI total score and executive function score were only found correlated with tau SUVR or volume of the hippocampal subfields with the presence of elevated amyloid pathology, which indicates that the decline of behavior and executive function is consistent with the neurodegenerative alterations of the HF when the subject was in the Alzheimer’s pathologic process.

Volume changes of the hippocampal subfields are not as regular as the deposition of tau tangles. The CA1, DG, and Sub of both hemispheres have a volume decrease in the A+ group as compared to the A− group, while no significant difference is found in the ERC, PHC, and CA2/CA3 (Table 2). There is nearly no significant association between the volume of the hippocampal subfields and assessment scores (except for the memory score) for the A- subjects (Table 4). With the presence of amyloid pathology, the relationship between the volume of the hippocampal subfields and assessment score is in conformity (Table 6). One possibility is that the volumetric changes of the hippocampal subfields are the sum of influences from normal aging and diverse neurodegenerative disorders such as AD, PD, and depression (Knopman et al., 2019). Individuals with diverse neuropsychiatric disorders may also be diagnosed as MCI or AD, which may affect the statistical results in the current study. Using volume changes of brain region without the help of AD-specific biomarkers is insufficient for the accurate diagnosis of AD (Edmonds et al., 2016; Sørensen et al., 2017).

Hemispheric difference is a classic topic in the neuroimaging area and still possesses a vital debate (Toga and Thompson, 2003; Pedraza et al., 2004; Woolard and Heckers, 2012). The left hippocampus was found more impaired than the right for individuals with subjective cognitive decline (Yue et al., 2018), as well as for the MCI and AD subjects (Shi et al., 2009). Similar results are discovered in the current research with the CA1 (*p* = 0.0069), DG (*p* = 0.0026), PHC (*p* = 0.0026), and CA2/CA3 (*p* = 1.92e-08) regions showing lower volume for the A− individuals. Lower volume of the left PHC (*p* = 0.0055) and CA2/CA3 (*p* = 2.57e-08) regions are also found for A+ group. In addition, the asymmetry of the association patterns between the tau SUVR of the hippocampal subfields and the assessment scores are obviously observed, and particularly more prominent for the NPI total score with the presence of elevated amyloid pathology (Table 5). Behavior symptoms including apathy, anxiety, and sleeping problems may be related to diverse brain functions and changes of specific hippocampal subfields (Chen et al., 2018; Campabadal et al., 2019; Dalton et al., 2019). The leftward asymmetry in the current research shows that the progression of AD may seriously affect the left hippocampi as compared to the right hemisphere.

## Limitations

The diagnosis of the patients from ADNI is based on the clinical symptoms and clinical assessment scores other than the amyloid PET or tau PET scans. This is why one AD patient and some MCI individuals are included in the A- group, while many CN subjects are found in the A+ groups. Considering the integrity of the original data as well as the relatively small sample size, we put the CN, MCI, and AD subjects together for the statistical analysis in the current study. It is insufficient to observe the alterations of tau deposition or volume changes of the hippocampal subfields during a specific stage of AD (normal aging, mild cognitive impairment, or AD) based on the limited data. This is one of the reasons why the association between the volume of hippocampal subfields and assessment scores show no consistent patterns as compared to the tau deposition. In addition, there is a significant difference of age between the A− and A+ groups (Table 1). However, the influence of normal aging is not demonstrated thoroughly even if we considered age as a covariance in the partial correlation process. On the other hand, NPI has 12 domains to assess the behavior symptoms of each subject and is widely used for the assessment of treatment effect (Cummings, 1997). However, the sample size of the current study is insufficient for the analysis of one specific behavior domain. Further analysis focused on the cohort with the same clinical diagnosis, as well as with the matched distribution of age, gender, education, etc., should be conducted with the development of the ADNI project.

Another limitation is that the relatively low resolution of the PET images may influence the calculation of the tau deposition for each hippocampal subfield. No partial volume correction was performed in this study, which may introduce signal mixture to small brain regions (Brendel et al., 2015; Matsubara et al., 2016; Rullmann et al., 2016; Su et al., 2016; Baker et al., 2017; Gonzalez-Escamilla et al., 2017). This is one of the reasons why the CA2 and CA3 were considered together and relatively large regions such as the CA1, ERC, and PHC were considered in the statistical analysis.

Not enough longitudinal data of the high-resolution T2 weighted MR images and tau PET scans for the statistical analysis were acquired based on the current inclusion criteria. AD is a neurodegenerative disorder that may go through several decades before clinical diagnosis. Observation of the dynamic changes of the tau deposition of the hippocampal subfields and its association with assessment scores in the future is essential for the early diagnosis and prevention of AD dementia.

## Conclusion

The current research has found that the development of AD may be affected in a progressive manner from the ERC to the PHC, the Sub, and eventually to the CA1 region on a smaller scale. The relationship between the clinical symptoms and tauopathy/volume of the hippocampal subfield showed diverse patterns under different amyloid statuses. Leftward asymmetry was observed with the association between assessment scores and tauopathy/volume of the hippocampal subfield, which is more prominent for the NPI total score in the current study. The associations of tauopathy/volume of the hippocampal subfields with the clinical symptoms are essential for the understanding of the AD spectrum and can be used as the reference for future AD studies.

## Data Availability Statement

The datasets presented in this study can be found in online repositories. The names of the repository/repositories and accession number(s) can be found in the article.

## Ethics Statement

Written informed consent was obtained from the individual(s) for the publication of any potentially identifiable images or data included in this article.

## Author Contributions

XG wrote the first draft of the manuscript. YZ and JX contributed to conception and design of the study. YQ and JZ performed the statistical analysis. XG and DZ organized the database and designed the protocol. All authors contributed to the article and approved the submitted version.

## Conflict of Interest

The authors declare that the research was conducted in the absence of any commercial or financial relationships that could be construed as a potential conflict of interest.

## Footnotes

^1^http://adni.loni.usc.edu/methods/documents/mri-protocols

^2^http://picsl.upenn.edu/software/ashs/

^3^https://ida.loni.usc.edu/
